# Innovative development of robot reduction system in geriatric pelvic fractures: A single-center case series in Beijing, China

**DOI:** 10.1016/j.jot.2024.08.023

**Published:** 2024-10-30

**Authors:** Chunpeng Zhao, Honghu Xiao, Qiyong Cao, Yufeng Ge, Yuneng Li, Yu Wang, Gang Zhu, Xinbao Wu

**Affiliations:** a Department of Orthopaedics and Traumatology, Beijing Jishuitan Hospital, Capital Medical University, Beijing, China; b School of Biological Science and Medical Engineering, Beihang University, Beijing, China; c Beijing Advanced Innovation Center for Biomedical Engineering, Beihang University, Beijing, China; d Rossum Robot Co., Ltd., Beijing, China

**Keywords:** Fragility, Minimal invasion, Pelvic fracture, Reduction, Robot

## Abstract

Displaced fragility fractures of the pelvis (FFP) pose significant challenges in orthopaedic trauma, owing to patient comorbidities, deteriorating bone quality, and surgical complexities. Despite technological advancements, no robotic methods have been documented for displaced FFP management. To address this, we introduced an advanced robot-assisted fracture reduction system, comprising a tracking device, path planning software, and robotic arms. This study evaluated fifteen consecutive patients with displaced FFP (average age 80.4 ± 9.1 years), who underwent robot-assisted reduction and internal fixation (RARIF) between January 2022 and May 2023. All were categorized as Rommens FFP type III, with a median time of 6 days (range 4–11) from injury to surgery. Operative times averaged 165 ± 44 min, with median blood loss of 50 mL. Postoperative radiographs showed all patients achieved excellent or good reductions as per Matta criteria, marking a 100 % success rate. A 6-month follow-up revealed an average modified Majeed score of 81.4, with 85.7 % of patients rated excellent or good. All fractures healed without complications. Leveraging our intelligent system, RARIF proves to be a safe and effective approach for displaced FFP, facilitating postoperative pain alleviation and early mobilization despite compromised health and bone conditions. This approach may revolutionize the management of FFP in an increasingly aging population, signaling a significant shift in therapeutic strategies.

Translational Potential of this Article: Elderly patients with displaced FFP often present complex surgical challenges due to comorbidities and poor bone quality, complicating reduction procedures and often leading to ineffective fixation. Addressing these challenges, we have developed an innovative robot-assisted fracture reduction system, offering a practical alternative to conventional methods. This system optimizes the applied force and direction during the reduction process, thereby reducing the needs for manual and repetitive attempts. Our report, detailing the successful implementation of this technique in 15 FFP cases, signifies a considerable leap forward in the field of orthopaedic surgery. This technique notably benefits the elderly population, a group traditionally marginalized in receiving care for complex orthopedic conditions.

## Graphical abstract

**Figure:**
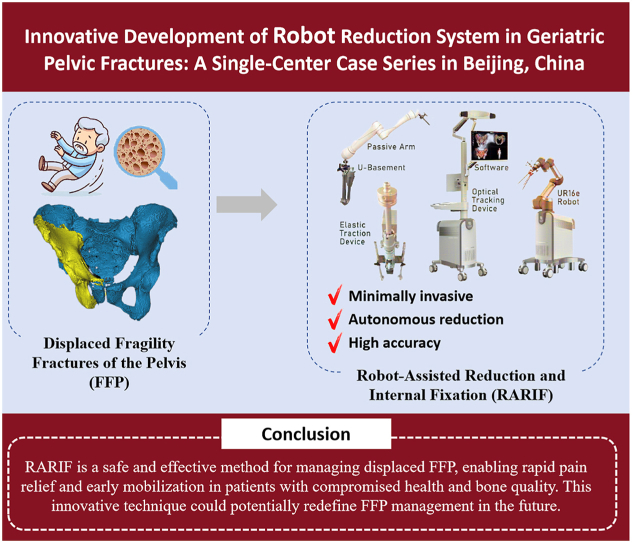

## 1 Introduction

Fragility fractures of the pelvis (FFP) typically result from low-energy trauma, posing a considerable risk of morbidity and mortality, especially in those with pre-existing medical conditions or functional limitations [1]. Osteoporosis is the primary cause of FFP, accounting for 7 % of osteoporotic fractures within the pelvic ring, and it is found that 94 % of individuals over 60 years old with pelvic fractures are osteoporotic [2,3]. The consequences of FFP—chronic pain, disability, and an increased risk of premature death—significantly contribute to the global health burden [4].

In treating pelvic fractures in elderly patients, where fragile bone structure and reduced physical condition are prevalent, the objectives focus on maintaining mobility and minimizing the risks associated with prolonged immobility [5]. Closed reduction and percutaneous internal fixation emerge as viable options, balancing minimal invasiveness with adequate fixation strength [[6], [7], [8]]. Nevertheless, performing these procedures requires a high level of expertise, particularly in elderly patients, where compromised bone quality limits the opportunity for repeated attempts. This poses a significant challenge to orthopedists worldwide. The advancement of computer technology in surgery, emphasizing minimally invasive techniques and accuracy, such as robotic arthroplasty and computer-assisted screw fixation [9,10], offers a potential solution to the difficulties of managing FFP. Researchers have previously introduced a computer-aided reduction frame based on the Starr frame [11], which still relies on the surgeon's hands to reduce the fragments. To date, no intelligent, robot-assisted techniques for displaced FFP have been documented.

In response, our team has developed an innovative, intelligent robot-assisted fracture reduction system for pelvic fractures, employing real-time navigation to enhance internal fixation accuracy [[12], [13], [14]]. This case series presents 15 displaced FFP cases to evaluate the safety and efficacy of our robot-assisted reduction and internal fixation techniques. To the best of our knowledge, this represents the first series of FFP cases managed using a robot-assisted approach for the reduction process.

## 2 Method

Between January 2022 and May 2023, we prospectively collected data on patients at our Beijing institution who underwent robot-assisted reduction and internal fixation (RARIF) for pelvic fractures, including their demographic and clinical characteristics. For the purposes in our study, we consecutively selected patients based on the FFP diagnostic criteria established by Rommens [15]. Exclusions were made for those with multiple traumatic injuries or who opted out of follow-up. This study received ethical approval from the Medical Ethics Committee of our institution (Approval No. 202006–13), and informed consent was obtained from all participants.

### 2.1 Robot-assisted fracture reduction system

This intelligent system for pelvic fracture reduction integrates three key elements (Fig. 1): pelvic reduction software that autonomously plans the reduction pathways to align the fragments with the targeted position, an NDI Polaris Vega optical tracking device for three-dimensional (3D) real-time visualization of the fracture pattern, and a UR16e robotic arm that completes the reduction procedure. Additionally, the system includes a passive gripping mechanism near the operating table and an elastic traction device at the end of the bed. The gripping mechanism features two 9-degree-of-freedom electric passive arms that secure the unaffected side of the pelvis to the table via a U-shaped connector. The traction device, powered by an electric screw, applies 0–30 kg of force to alleviate soft tissue constraints and reduce the load on the robotic arm [16].

**Figure 1:**
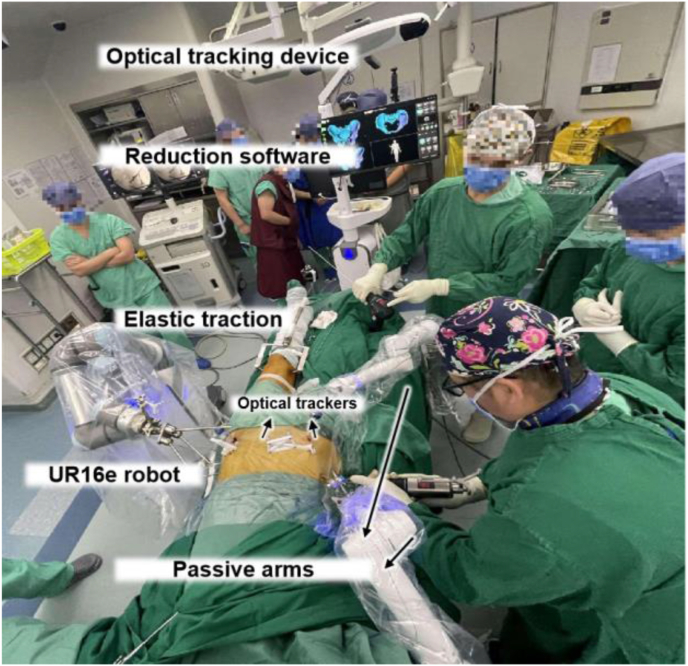
Structure and spatial location of the robot system in the operating room.

**Figure 2:**
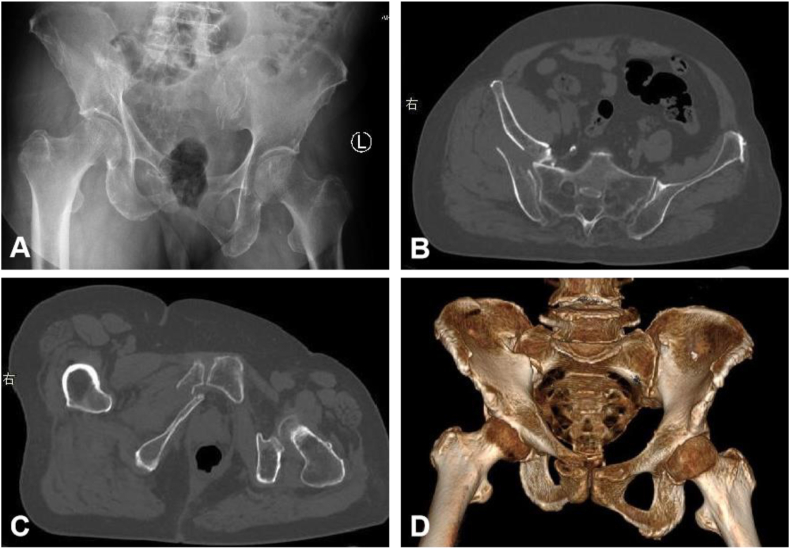
Image of the displaced pelvic fracture in a 83-year-old female with heart disease and severe osteoporosis (A: anterioposterior view; B–C: axial CT view of fractured posterior and anterior ring; D: 3D reconstruction view).

**Figure 3:**
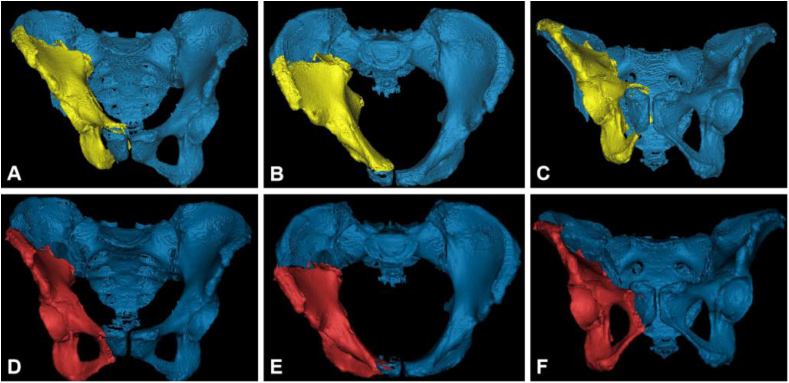
Position of fragments before and after autonomous reduction planning (A–C: original position; D–F: final target position).

Before surgery, patients undergo 3D CT scans of the pelvis, which are analyzed by the reduction planning software to identify fracture fragments and design a tailored reduction path aimed at achieving pelvic symmetry (Figure 2, Figure 3). This strategy provides surgeons with the flexibility to dynamically adjust the procedure, setting waypoints to prevent collisions, torsion, or blockages between fragments during the reduction process.

### 2.2 Surgical procedure

*Perioperative care:* Before surgery, all patients received antithrombotic prophylaxis, and contraindications were excluded. A multidisciplinary team was available if necessary. After surgery, patient-controlled analgesia was used. All patients received rehabilitation consultation and disease-related education before discharge.

*Tracker Placement and Imaging:* Patients, under general anesthesia, were positioned supine on a radiolucent surgical bed with their buttocks elevated. Trackers were attached to the anterior superior iliac spines on both sides. Pelvic and tracker data were then acquired using a Cone-beam CT (CBCT) scan with a Siemens C-arm X-ray machine. This CBCT data was sent to the computer system for image merging with the preoperative 3D CT scan. Navigation software aligned the high-resolution CT images with the CBCT, enabling real-time pelvic tracking displayed onscreen.

*Reduction and Traction Setup:* Guided by a real-time hand drill navigation system, 5.0 mm Schanz pins were inserted into the pelvis on both sides. Gripping pins were precisely placed at the iliac crest (gluteal tuberosity) and the anterior inferior iliac spine. Additionally, a horizontal pin was positioned above the acetabulum on the affected side. Two passive arms were then connected to the pins on the healthy side, and a robotic arm was attached to the affected side. Supracondylar bone traction was applied to patients experiencing vertical displacement of pelvic fractures, utilizing elastic traction devices for connection. The traction force was dynamically adjusted based on the alterations observed in the 3D navigational images post-registration. Once set, this traction force was maintained constant throughout the surgical procedure.

*Automated Reduction:* Directed by 3D real-time navigation, the robotic arm automatically moved the affected pelvic half along the predetermined path (Supplement video S1). Should excessive resistance occur, the system enabled an auxiliary force mode, permitting manual assistance with the robotic arm until correct alignment was achieved.

**Figure 4:**
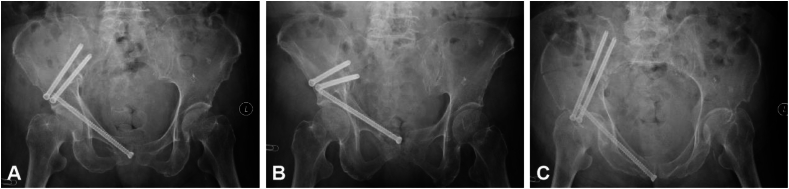
Postoperative radiographs to evaluate the reduction quality.

*Position Verification and Fixation:* Following reduction, the robotic arm secured the pelvic position. Fluoroscopy was utilized to confirm fracture reduction adequacy (Fig. 4), cross-referenced with 3D navigational images. Depending on the fracture's nature, stabilization was achieved through percutaneous channel screws, external fixation frames, or internal pelvic fixators (INFIX). Standard anterior-posterior and inlet-outlet pelvic radiographs were obtained to verify the reduction's success and ensure accurate screw placement before surgery completion.

### 2.3 Data collection and follow-up

Radiological outcomes were evaluated using postoperative CT scans and plain radiographs across three standard pelvic views: antero-posterior, inlet, and outlet. Reduction quality was assessed based on the Matta criteria [17]. Preoperative planned 3D models and actual postoperative CT scans of the pelvis were analyzed using Geomagic Qualify 2013 software (Geomagic Inc, USA). This analysis involved calculating the global 3D point cloud error to compare preoperative plans with postoperative results, recording the mean of all absolute deviation values as the reduction error.

Follow-up procedures were standardized, with all patients undergoing clinical evaluations at 6 weeks, 3 months, and 6 months after surgery. These follow-ups included determining the all-cause mortality rate, interviewing surviving patients, and using X-ray imaging to assess fracture healing. Observations were made for signs of infection, implant loosening, displacement, or re-fracture to assess the procedure's safety. At the final follow-up, functional outcomes were measured using a modified Majeed Score, adjusted to a 100-point scale by excluding work and sexual intercourse aspects (Total = sum/76 × 100).

Data presentation involves means ± standard deviations for normally distributed parametric data and medians with interquartile ranges for non-normally distributed data. Categorical variables are presented as frequencies and percentages.

## 3 Result

The study population consisted of 4 men and 11 women, with an average age of 80.4 ± 9.1 years (range, 65–92 years). The average Body Mass Index (BMI) was 22.46 ± 3.34 (range, 15.6–27). All pelvic fractures in the study were classified as closed. Of the participants, 9 had two or more comorbidities, including heart or lung disease, cerebrovascular disease, and diabetes. The majority, 11 patients (73.3 %), were categorized as ASA class III. According to Rommens’ criteria, all patients had type III FFP fractures, with surgery occurring a median of 6 days (range 4–11 days) post-injury. Patient demographics and clinical characteristics are detailed in Table 1.

**Table 1:** Patients’ demographics and operative data.

NO	Age (years)	Gender	BMI	FFP classification/AO classification	Heart Diseases	Diabetes Mellitus	ASA	Time from injury to surgery (days)	Operative time (min)	Blood loss (ml)	Matta's quality of reduction	Reduction Error	Postoperative LOS (days)
1	65	Female	24.7	IIIa/C1	0	0	2	18	225	200	1	4.4	3
2	71	Female	18.9	IIIa/C1	0	0	3	15	175	100	1	3.3	13
3	83	Female	27	IIIa/C1	1	0	3	3	125	100	2	4.7	5
4	84	Female	22.6	IIIa/B2	0	0	3	20	170	20	2	2.4	3
5	85	Female	20.2	IIIa/C1	0	1	2	13	120	50	2	4.9	4
6	86	Female	22.2	IIIa/C1	1	1	3	3	180	100	2	4	4
7	89	Female	22.9	IIIa/B2	1	0	3	9	190	50	2	4.3	3
8	89	Male	22.9	IIIa/B2	1	1	3	9	150	50	1	3.7	3
9	92	Female	26.9	IIIb/C1	1	0	3	1	120	10	2	1.8	5
10	67	Male	26.4	IIIc/C1	1	0	3	6	165	50	2	7.9	5
11	86	Female	17.7	IIIa/B2	1	0	3	4	270	50	2	3.1	2
12	71	Male	21.2	IIIa/C1	0	0	1	8	105	20	2	8.5	5
13	78	Female	24.7	IIIa/C1	1	1	3	6	135	20	1	2.9	5
14	71	Male	23.0	IIIb/C1	0	0	1	5	195	50	2	5.5	3
15	89	Female	15.6	IIIa/C1	1	0	3	4	150	20	1	4.7	5

Abbreviations: *BMI* bone mass index, *FFP* fragility fractures of the pelvis, *AO* Arbeitsgemeinschaft für Osteosythese, *ASA* American society of anesthesiologists, *LOS* length of stay;

Operative times averaged 165 ± 44 min (range, 105–270 min), with median intraoperative blood loss recorded at 50 mL (range, 10–200 mL). The mean residual displacement was 5.4 ± 2.7 mm (range, 1.1–9.7 mm). Postoperative radiographs indicated that all patients achieved reductions rated as excellent or good by Matta criteria, resulting in a 100 % success rate. The average discrepancy between preoperative plans and postoperative outcomes, measured by the global 3D point cloud reduction error, was 4.4 ± 1.8 mm (range, 1.8–8.5 mm). The average postoperative hospital stay was 4.5 ± 2.6 days (range, 2–13 days).

At a 6-month postoperative follow-up, fourteen patients were evaluated while one had passed away due to heart disease. Detailed outcomes are presented in Table 2. The average modified Majeed score was 81.4 ± 10.2, with 85.7 % of patients receiving excellent or good ratings. All fractures healed without any complications. A typical case was demonstrated in Supplement Fig. 1.

**Table 2:** Outcomes of six-month follow-up.

NO	Majeed Scoring system							Survival
	Pain	Sitting	Walking aids	Gait unaided	Walking distance	Total^a^	Grade	
1	30	10	10	6	10	86.8	Excellent	Yes
2	30	10	10	6	10	86.8	Excellent	Yes
3	30	10	8	0	8	73.7	Good	Yes
4	30	10	12	12	12	100	Excellent	Yes
5	30	10	12	4	6	81.6	Good	Yes
6	30	10	10	4	10	84.2	Good	Yes
7	30	10	10	6	10	86.8	Excellent	Yes
8	30	10	8	4	8	78.9	Good	Yes
9	30	10	10	6	10	86.8	Excellent	Yes
10	25	8	12	12	12	90.8	Excellent	Yes
11	30	10	4	4	4	68.4	Fair	Yes
12	20	6	10	12	8	73.7	Good	Yes
13	15	6	10	8	6	59.2	Fair	Yes
14	30	8	12	8	4	81.6	Good	Yes
15	—	—	—	—	—	—	—	No

a Excluding work and sexual intercourse aspects, the modified Majeed score is adjusted to a 100-point scale (Total = sum/76 × 100).

## 4 Discussion

Recent advancements in pelvic fracture surgery have increasingly leveraged robotic and computer-assisted technologies, exemplified by systems such as the TiRobot system (TINAVI Medical Technologies, China) [12,18,19]. These innovations have proven exceptionally beneficial in treating FFP, where navigated minimally invasive percutaneous fixation techniques have reduced stress on elderly patients and enhanced success rates [20]. Despite these advancements, the development of intelligent technologies specifically designed for the reduction of pelvic fractures remains limited. This study showcases 15 cases that illustrate the successful application of a novel robotic technology in the reduction of displaced FFP, underscoring its substantial potential for wider implementation.

The pelvic ring's unique anatomical configuration, comprising both anterior and posterior segments, presents specific reduction challenges due to its irregular shape and deep location. Often, reduction may seem satisfactory from one perspective, yet from another, it reveals displacement. Consequently, the techniques typically used for long bone fractures are insufficient for pelvic fracture reduction, leading to a high incidence of failure in closed reduction attempts, even among experienced trauma orthopedic surgeons.

FFP fractures, predominantly affecting older adults, further complicate the management of pelvic surgeries [21]. This complexity arises from two main factors: first, the limited capacity of these patients to withstand the trauma and bleeding associated with conventional open reduction methods, due to frailty and comorbidities; second, their generally poor bone quality resulting from osteoporosis [22]. This scenario closely parallels the difficulties encountered in managing fragility hip fractures in the elderly, where osteoporosis is also the main cause. Osteoporosis substantially increases the risk of closed reduction and fixation efforts' failure [23]. Manual attempts at closed reduction often necessitate a shift to open reduction, heightening the risk of additional trauma and surgical failure. In 2016, inspired by Lefaivre's Starr frame [24], Zhang et al. [25] introduced a computer-aided technique that uses software and an intraoperative 3D reconstruction model to calculate the residual differences between the real-time and targeted reduction positions. However, the reduction accuracy depended on the surgeon and required multiple adjustments and attempts. After each movement, an intraoperative CT scan or fluoroscopy was needed to verify the results, raising concerns about excessive radiation exposure. In our study, we introduced a technique that not only provides a minimally invasive solution but also uses comprehensive real-time 3D navigation to intelligently design a targeted reduction pathway. This approach circumvents the risks associated with manual, unguided attempts at reduction, effectively addressing the challenges outlined above.

The application of artificial intelligence technology in facilitating reduction efforts introduces the challenge of precisely determining placement after reduction. Our research utilized the mirroring principle of the contralateral healthy pelvic side, combined with 3D imaging, to simulate the expected post-reduction fracture placement [26]. Through intelligent planning of the reduction path and precise calculation of the optimal force magnitude and direction, the system fine-tunes the application of combined force. This approach significantly enhances the accuracy and success rate of pelvic fracture reduction. The methodical, gradual, and intentional nature of the entire reduction process not only increases the success rate but also minimizes the risk of causing additional damage, such as cutting through osteoporotic bone due to force dispersion [27]. No surgery-related complications were observed in our series, demonstrating the preliminary safety of our RARIF technique.

To our knowledge, this study represents the first case series employing robotic-assisted reduction and internal fixation within the FFP demographic. Our findings suggest that this technique is both safe and effective for this distinct patient population. Nevertheless, there are several limitations to consider. Firstly, the small sample size and the absence of a comparative analysis with traditional methods restrict further evaluation of its effectiveness, inherently reducing the level of evidence characteristic of case series studies. Secondly, given our study's focus on assessing the effectiveness of our technique in FFP, we conducted only a six-month postoperative follow-up to observe early functional recovery, which constrains our capacity to assess long-term outcomes. Future research, ideally in the form of a prospective randomized controlled trial featuring a robust design and extended follow-up period, is necessary to validate the superiority of our technique over conventional surgical approaches in managing FFP.

In conclusion, our study's findings indicate the safety and effectiveness of the RARIF approach in managing displaced FFP, enabling postoperative pain relief and early mobilization even in patients with compromised health and bone quality. Such an innovative technique has the potential to transform FFP management in an aging population, marking a pivotal shift in treatment strategies. Future comparative research is necessary to further explore the indications for our intelligent technique and its advantages over traditional methods.

## Funding

This work was supported in part by the 10.13039/501100001809National Natural Science Foundation of China (Grant no. 61871019), the 10.13039/501100012401Beijing science and technology project (Z201100005420033), the Beijing Jishuitan Research Funding (YGQ-202309), and the 10.13039/501100004826Natural Science Foundation of Beijing (19L2011).

## Author contributions

Conceptualization: C.Z., H.X., Y.W. Y.L. and X.W.; Data curation: H.X., Q.C., Y.G., G.Z., and X.W.; Formal analysis, investigation and methodology: C.Z., Y.G., Y.W., and H.X.; Funding acquisition: C.Z., Y.L., and X.W.; Supervision: Y.W., and X.W.; Writing-original draft: C.Z., H.X., Y.G. and G.Z.; Writing-review & editing: Y.G., Q.C., Y.W., Y.L., and X.W.; All authors have read and agreed to the published version of the manuscript.

## Ethics approval and consent to participate

Approval was obtained from the institutional review boards of the Jishuitan Hospital, Beijing, China, and all procedures adhered to the tenets of the Declaration of Helsinki (Approval no.: 202,006–13)). Informed consent was obtained from all individual participants included in the study.

## Data availability

The datasets used in this study are not publicly available because of participant confidentiality but are available from the corresponding author on reasonable request.

## Declaration of competing interest

The authors declare no conflicts of interest.

## Figures and Tables

**figs1:**
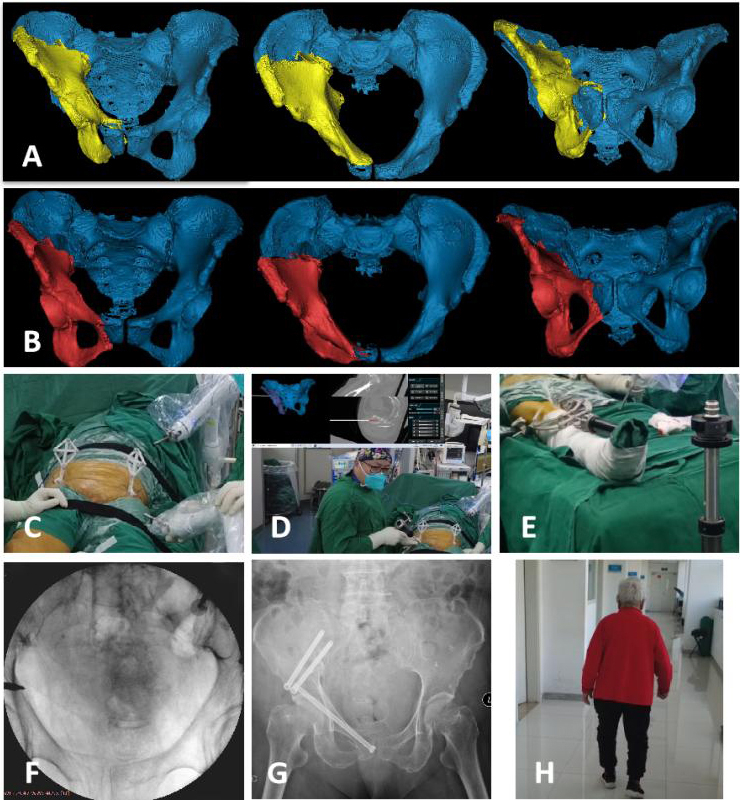
Supplementary Fig.1 Typical case. A 78-year-old female patient with a Tile type C1 pelvic fracture from a traffic accident underwent robotassisted closed reduction and internal fixation five days post-admission. Reduction path was autonomously planned based on preoperative reconstructed CT image (A-B). Installation of trackers on both pelvic rings (C), followed by CBCT scanning and image registration, preceded the placement of Schantz pins and the attachment of passive holding arms and the robot arm to the respective sides of the pelvis (D). An elastic traction system was installed on the femur's supracondylar region (E). The quality of the reduction was verified intraoperatively with fluoroscopy (F), and a percutaneous screw was inserted for fixation. Postoperative X-rays confirmed decent fracture reduction (G). Twelve weeks postsurgery, the patient regained pre-injury mobility (H).

## Appendix A Supplementary data

Multimedia component 1 Supplementary Video 1. Intra-operative autonomous reduction procedure. Multimedia component 1
